# Gradual Dislocation of the Femur, from Disease

**Published:** 1860-07

**Authors:** H. D. Adams

**Affiliations:** Union Grove


ARTICLE 5.

### GRADUAL DISLOCATION OF THE FEMUR FROM DISEASE.

UNION GROVE, April 23, 1860.

PROF. BRAINARD—Sir: As there is a case of some interest at present in the Racine County Poor House, I thought I would state the history and present appearance of the patient, and, if convenient, would like your opinion.

The subject is an African, about thirty years old, a strong, muscular man. He says that about fifteen years ago he was attacked with rheumatism in his left hip, which continued, with more or less severity, for three years, from which time till the present he has been unable to use his left limb, or to get about, except with a crutch.

Present appearance: General health good; the left limb much smaller than the other; length of limb the same, from foot to knee and from knee to head of femur, as the other; the head of the femur is removed from its socket, upwards and backwards, a trifle over three inches; the foot is drawn across the instep of the right foot, toes turning inwards; the knee of the left leg rests on the anterior of the right knee. There is no swelling of the hip, but some pain on moving it. By taking hold of the leg it can be moved in all directions, but not so readily backwards as in other directions. The above has been the condition of the leg for the last twelve years. He is not conscious of ever receiving any injury, or suffering from any disease except rheumatism.

He is anxious something should be done, if possible to relieve his present situation.

I state this case in hopes to obtain your opinion of it.

Yours, &c.,
H. D. ADAMS.

---

*Remarks.*—Spontaneous dislocation of the hip, or gradual dislocation, from disease, is an accident the occurrence of which has recently been called in question, particularly by Prof. Alden March, of Albany. The above seems to be a case of the kind. We have, in the cabinet of Rush Medical College, three specimens showing this species of accident, besides three more in which the nature of the accident is less certain, but they are, probably, cases of this kind.

We know of no treatment likely to be of service in relieving this deformity.

